# Supplementary material for: Understanding dimensions of trust in AI through quantitative cognition: Implications for human-AI collaboration
Source: PLoS One. 2025 Jul 2;20(7):e0326558. doi: 10.1371/journal.pone.0326558 (PMC12221052; doi:10.1371/journal.pone.0326558)
Supplement: S1 Table — (DOC) [file pone.0326558.s001.doc]

# Supporting information

**S1 Table. Summary of Trust in AI research literature**.

| **Study** | **Trust Type** | **Dimension(s)** | **Related Constructs** | **Method(s)** | **Key Relationships and Findings** |
| --- | --- | --- | --- | --- | --- |
| Kim et al. (2021) | Human-like | Benevolence, Reliability | Customer Satisfaction | One-factor, between-group experimental design | The accuracy and reliability of information affects behaviour and evaluation orientation, but extrapolation of conclusions is limited by the lack of diversity of variable indicators. |
| Gil et al. (2019) | Human-like | Anthropomorphism | Interaction, Control, Intelligibility | Case study | Anthropomorphic design improves human-AI interaction acceptance through intuitive control. |
| Califf et al. (2020) | Human-like | Anthropomorphism | Social cognition, Trust comparison | Questionnaire | Human-like systems have a greater effect on perceived usefulness, and systemic versus interpersonal trust has a differential effect on platform acceptance. |
| Jung et al. (2019) | Human-like | Risk-bearing | Social influence, Emotional reaction | Cognitive neuroscience experiments | Human-like AI evokes differentiated neural responses; social context remains underexplored. |
| Choung et al. (2022) | Human-like & Cognitive | Fairness, Embodiment, Empathy, Tangibility | TAM constructs | Questionnaire | Perceived fairness and embodiment impact acceptance; limitations in sample and tech types. |
| Choung et al. (2023) | Functionality | Reliability | Practicality | Online Questionnaire | Functionality-driven trust increases when perceived usefulness is high. |
| Habbal et al. (2024) | Functionality | Predictability | Risk management | Theory and case studies | AI TRiSM demands trust based on predictability; empirical work is lacking. |
| Pereira et al. (2023) | Functionality | Transparency | Openness, Access | Theory and case studies | Transparent AI systems require normative framing; few studies address practical guidance. |
| Huang & Rust (2021, 2024) | Functionality | Feedback & Evaluation | Past interaction memory | Theory and case studies | The functions of the three types of AI (functional trust) (mechanical, thinking, and feeling) are proposed, but empirical testing is lacking. |
| Okamura & Yamada (2020) | Functionality | Mission Characteristics | Environment-task-AI fit | Experiments | Task-agent matching influences trust calibration effects, but there are trust model as well as insufficient participant diversity limitations. |
| Georganta & Ulfert (2024) | Cognitive | Reliability | Team dynamics, Collaboration | Questionnaires and experiments | Coherent and consistent performance enhances team trust in AI members, but AI diversity is understudied. |
| Bedué & Fritzsche (2022) | Cognitive | Capacity | Corporate AI adoption | Interviews and surveys | AI capability perception (knowledge of AI) boosts adoption intentions, but lacks quantitative validation. |
| Buçinca et al. (2021) | Cognitive | Transparency | Overreliance, Dual-process reasoning | Experiments | Transparency reduces reliance on AI through cognitive forcing, but lacks cross-industry considerations. |
| Chi et al. (2021) | Emotional | Social/Cultural | Trust propensity, Human factors | Questionnaires and experiments | Different cultural backgrounds affect the level of human-computer interaction and emotional support; experimental limitations |
| Riley & Dixon (2024) | Emotional | Emotional support | Human emotion recognition | Theory and case studies | There is a gap in empathy-driven AI (emotional) trust research and empirical expansion is needed |
