# Supplementary material for: Understanding dimensions of trust in AI through quantitative cognition: Implications for human-AI collaboration
Source: PLoS One. 2025 Jul 2;20(7):e0326558. doi: 10.1371/journal.pone.0326558 (PMC12221052; doi:10.1371/journal.pone.0326558)
Supplement: S2 Table — (DOC) [file pone.0326558.s002.doc]

# Supporting information

**S2 Table. Reliability, validity, and convergence analysis of Trust in AI questionnaire items.**

| **Secondary Concepts** | **Items** | **KMO** | **Total Variance Explanation** | **AVE** | **CR** |
| --- | --- | --- | --- | --- | --- |
| **Human-like trust** | HLT1 | 0.82 | 73% | 0.73 | 0.92 |
|  | HLT2 |  |  |  |  |
|  | HLT3 |  |  |  |  |
|  | HLT4 |  |  |  |  |
| **Functionality trust** | FT1 | 0.74 | 80% | 0.81 | 0.93 |
|  | FT2 |  |  |  |  |
|  | FT3 |  |  |  |  |
| **Cognitive trust** | CT1 | 0.74 | 60% | 0.6 | 0.82 |
|  | CT2 |  |  |  |  |
|  | CT3 |  |  |  |  |
|  | CT4 |  |  |  |  |
| **Emotional trust** | ET1 | 0.71 | 78% | 0.78 | 0.91 |
|  | ET2 |  |  |  |  |
|  | ET3 |  |  |  |  |
