# Supplementary material for: Understanding dimensions of trust in AI through quantitative cognition: Implications for human-AI collaboration
Source: PLoS One. 2025 Jul 2;20(7):e0326558. doi: 10.1371/journal.pone.0326558 (PMC12221052; doi:10.1371/journal.pone.0326558)
Supplement: S3 Table — (DOC) [file pone.0326558.s003.doc]

# Supporting information

**S3. Table Quantitative cognitive.**

| **Items** | **Test-Design** | **Reference** | **Criteria** | **Define** |
| --- | --- | --- | --- | --- |
| **Rem** | List commonly used models and equations | listing | Based on remembering what was learned | Information recall accuracy |
|  | Questionnaire Design Structure and Notes | Retrieving/ Identifying |  |  |
| **Und** | List at least 3 ways to survey data and describe the scope of application | Summarising/ Inferring | Depending on the understanding of the different approaches | Decoding of information |
|  | Compare the advantages and disadvantages of different analytical methods, their corresponding data types and usage Scenarios | Comparing/ Explaining |  |  |
|  | Listing of tools and websites on social networking, knowledge organization and data acquisition | Social networking/ Boolean Searching/ Categorising/ Subscribing | Proper selection and application of learning tools and components |  |
| **App** | Demo: How do you find data? What channels or tools were used to find the data | Using/ implementing/ showing | Rate ability to uncover and analyze data through presentations and demonstrations | Mission-specific applications |
|  | Demo: data processing; show the app or program used |  |  |  |
| **Ana** | Visual data presentation of automobiles and explaining the meaning behind the data | Organising/ Deconstructing | Rating based on match of scenarios, data types, analytics and insights | Breakdown and evaluation of information |
| **Eva** | Which partners gave me which advice regarding this conclusion? How did I interpret these suggestions (reflect on them)? | Collaborating/ networking | Rating of judgment and reflection based on comments on others' contributions | Assessment and judgement of information or programmes |
| **Cre** | Overall evaluation of data investigation, processing and conclusions | Directing/ producing | Rate the report for predictive, practical, instructive and creative | Innovative programmes |

Note: Rem=Remembering, Und=understanding, App=Applying, Ana=analyzing, Eva=evaluating, Cre=creating.
