# Supplementary material for: Understanding dimensions of trust in AI through quantitative cognition: Implications for human-AI collaboration
Source: PLoS One. 2025 Jul 2;20(7):e0326558. doi: 10.1371/journal.pone.0326558 (PMC12221052; doi:10.1371/journal.pone.0326558)
Supplement: S4 Table — (DOC) [file pone.0326558.s004.doc]

# Supporting information

**S4 Table. Regression results for 100 experiments with parameter tuning (learning rate: 0.01, iterations: 1000).**

| **variable** | **remember** | **understanding** | **application** | **analysis** | **evaluation** | **creating** |
| --- | --- | --- | --- | --- | --- | --- |
| **intercept** | 0.0060 | -0.0281 | -0.0323 | 0.0055 | 0.0304 | -0.0091 |
| **HLT1** | 0.0598 | 0.0061 | 0.0589 | 0.0362 | -0.0219 | 0.0613 |
| **HLT2** | 0.0135 | 0.1191 | 0.0312 | 0.0270 | 0.0365 | 0.0449 |
| **HLT3** | 0.0519 | 0.0770 | 0.0534 | -0.0110 | 0.0079 | 0.0068 |
| **HLT4** | 0.0101 | -0.0697 | -0.0280 | 0.0925 | 0.0530 | -0.0680 |
| **FT1** | 0.0900 | 0.0202 | 0.0284 | -0.0085 | 0.0801 | 0.0538 |
| **FT2** | 0.0901 | 0.1090 | 0.0690 | -0.0250 | 0.1167 | 0.0774 |
| **FT3** | 0.0197 | 0.0005 | 0.1152 | 0.1939 | 0.0727 | 0.1008 |
| **CT1** | -0.1519 | -0.1284 | -0.1095 | -0.0371 | -0.0600 | -0.0127 |
| **CT2** | -0.0167 | -0.0761 | -0.0091 | -0.1011 | 0.0279 | -0.0554 |
| **CT3** | -0.1143 | 0.0047 | -0.0203 | 0.0212 | -0.1749 | -0.0605 |
| **CT4** | 0.0377 | 0.0075 | 0.0078 | -0.0756 | -0.0452 | -0.0753 |
| **ET1** | 0.0713 | -0.0548 | 0.0706 | 0.0943 | 0.0539 | 0.0719 |
| **ET2** | -0.0380 | 0.0762 | 0.0057 | 0.1516 | 0.0570 | 0.1032 |
| **ET3** | -0.0105 | 0.0108 | 0.0730 | 0.0460 | 0.0725 | 0.0239 |

Note：learning rate of 0.01 and a number of iterations of 1000.
