# Supplementary material for: Understanding dimensions of trust in AI through quantitative cognition: Implications for human-AI collaboration
Source: PLoS One. 2025 Jul 2;20(7):e0326558. doi: 10.1371/journal.pone.0326558 (PMC12221052; doi:10.1371/journal.pone.0326558)
Supplement: S6 File — (DOC) [file pone.0326558.s006.doc]

**Mean TMSE Calculation for Parameter Tuning (400 Runs)**

data = readtable( );

X = data{:, {'HLT1', 'HLT2', 'HLT3', 'HLT4','FT1', 'FT2','FT3','CT1', 'CT2', 'CT3', 'CT4', 'ET1', 'ET2', 'ET3'}};

Y = data{:, {'remember', 'understanding', 'application','analysis', 'evaluation', 'creating'}};

[X, muX, sigmaX] = zscore(X);

[Y, muY, sigmaY] = zscore(Y);

num_repeats = 400;

alpha = 0.0001;

num_iterations = 5000;

[num_samples, num_features] = size(X);

num_outputs = size(Y, 2);

tmse_results = zeros(num_repeats, 1);

for rep = 1:num_repeats

cv = cvpartition(size(X, 1), 'HoldOut', 0.2);

idx = cv.test;

X_train = X(~idx, :);

Y_train = Y(~idx, :);

X_test = X(idx, :);

Y_test = Y(idx, :);

X_train = [ones(size(X_train, 1), 1) X_train];

X_test = [ones(size(X_test, 1), 1) X_test];

theta = zeros(num_features + 1, num_outputs);

for iter = 1:num_iterations

predictions = X_train * theta;

errors = predictions - Y_train;

gradients = (1 / size(X_train, 1)) * (X_train' * errors);

theta = theta - alpha * gradients;

end

predictions_test = X_test * theta;

errors_test = predictions_test - Y_test;

mse_test = (1 / (2 * size(X_test, 1))) * sum(errors_test .^ 2, 1);

total_mse_test = sum(mse_test);

tmse_results(rep) = total_mse_test;

end

fprintf('Repeated 400 times:\n');

fprintf('Mean TMSE: %.4f\n', mean(tmse_results));

fprintf('Std Dev TMSE: %.4f\n', std(tmse_results));

figure;

histogram(tmse_results, 15);

xlabel('Total MSE on Test Set');

ylabel('Frequency');

title('Distribution of TMSE over 400 Repeats');

grid on;

**Overfitting Trend (100 Iterations/Experiments)**

filePath = ' ';

data = readtable(filePath);

X = data{:, {'HLT1', 'HLT2', 'HLT3', 'HLT4', 'FT1', 'FT2', 'FT3', 'CT1', 'CT2', 'CT3', 'CT4', 'ET1', 'ET2', 'ET3'}};

Y = data{:, {'KC_remember', 'KC_understanding', 'KC_application', 'KC_analyzing', 'KC_evaluation', 'KC_creation'}};

[X, muX, sigmaX] = zscore(X);

[Y, muY, sigmaY] = zsc

hiddenLayerSize = 10;

num_trials = 100;

mse_all = zeros(num_trials, size(Y, 2));

for i = 1:num_trials

cv = cvpartition(size(X, 1), 'HoldOut', 0.2);

X_train = X(training(cv), :);

Y_train = Y(training(cv), :);

X_val = X(test(cv), :);

Y_val = Y(test(cv), :);

net = feedforwardnet(hiddenLayerSize);

net.trainParam.epochs = 5000;

net.trainParam.lr = 0.0001;

net.trainParam.showWindow = false;

net.trainParam.showCommandLine = false;

net = configure(net, X_train', Y_train');

[net, tr] = train(net, X_train', Y_train');

Y_pred = net(X_val');

mse_val = mean((Y_pred' - Y_val).^2);

mse_all(i, :) = mse_val;

end

avg_mse = mean(mse_all, 1);

std_mse = std(mse_all, 0, 1);

output_names = {'KC_remember', 'KC_understanding', 'KC_application', ...

'KC_analyzing', 'KC_evaluation', 'KC_creation'};

fprintf('Validation MSE (mean ± std) over %d trials:\n', num_trials);

for j = 1:length(output_names)

fprintf('%s: %.4f ± %.4f\n', output_names{j}, avg_mse(j), std_mse(j));

end

figure;

plotperform(tr);

title('Training Performance (Last Trial)');

**Loss Curve from 100 Runs**

data = readtable(' ‘);

X_all = data{:, {'HLT1', 'HLT2', 'HLT3', 'HLT4','FT1', 'FT2', 'FT3','CT1', 'CT2', 'CT3', 'CT4', 'ET1', 'ET2', 'ET3'}};

Y_all = data{:, {'KC_remember', 'KC_understanding', 'KC_application', 'KC_analyzing', 'KC_evaluation', 'KC_creation'}};

[X_all, muX, sigmaX] = zscore(X_all);

[Y_all, muY, sigmaY] = zscore(Y_all);

num_iterations = 1000;

num_runs = 100;

alpha = 0.01;

[num_samples, num_features] = size(X_all);

num_outputs = size(Y_all, 2);

mse_history_all = zeros(num_iterations, num_outputs);

for run = 1:num_runs

cv = cvpartition(num_samples, 'HoldOut', 0.2);

idx = cv.test;

X_train = X_all(~idx, :);

Y_train = Y_all(~idx, :);

X_test = X_all(idx, :);

Y_test = Y_all(idx, :);

X_train = [ones(size(X_train,1),1), X_train];

X_test = [ones(size(X_test,1),1), X_test];

theta = zeros(num_features + 1, num_outputs);

mse_history = zeros(num_iterations, num_outputs);

for iter = 1:num_iterations

predictions = X_train * theta;

errors = predictions - Y_train;

gradients = (1/size(X_train,1)) * (X_train' * errors);

theta = theta - alpha * gradients;

mse = (1/(2*size(X_train,1))) * sum(errors .^ 2, 1);

mse_history(iter, :) = mse;

end

mse_history_all = mse_history_all + mse_history;

end

mse_history_avg = mse_history_all / num_runs;

figure;

hold on;

output_names = {'KC\_remember', 'KC\_understanding', 'KC\_application', 'KC\_analyzing', 'KC\_evaluation', 'KC\_creation'};

for j = 1:num_outputs

plot(1:num_iterations, mse_history_avg(:, j), 'LineWidth', 2, 'DisplayName', output_names{j});

end

xlabel('Iterations');

ylabel('Average MSE');

title(['Average Loss Curve over ', num2str(num_runs), ' Runs']);

legend('show');

grid on;

hold off;

set(gcf, 'PaperUnits', 'inches', 'PaperPosition', [0 0 8 6]);

print(gcf, 'avg_loss_curve', '-dpng', '-r300'); avg_loss_curve.png

**Gradient Regression Coefficients (Averaged Over 100 Tests)**

data = readtable(' ');

X = data{:, {'HLT1', 'HLT2', 'HLT3', 'HLT4','FT1', 'FT2', 'FT3','CT1', 'CT2', 'CT3', 'CT4', 'ET1', 'ET2', 'ET3'}};

Y = data{:, {'KC_remember', 'KC_understanding', 'KC_application', 'KC_analyzing', 'KC_evaluation', 'KC_creation'}};

[X, muX, sigmaX] = zscore(X);

[Y, muY, sigmaY] = zscore(Y);

cv = cvpartition(size(X, 1), 'HoldOut', 0.2);

idx = cv.test;

X_train = X(~idx, :);

Y_train = Y(~idx, :);

X_test = X(idx, :);

Y_test = Y(idx, :);

[num_samples, num_features] = size(X_train);

num_outputs = size(Y_train, 2);

alpha = 0.01;

num_iterations = 500;

num_trials = 100;

X_train = [ones(num_samples, 1) X_train];

X_test = [ones(size(X_test, 1), 1) X_test];

mse_history_all = zeros(num_iterations, num_outputs, num_trials);

theta_all = zeros(num_features + 1, num_outputs, num_trials);

for trial = 1:num_trials

theta = zeros(num_features + 1, num_outputs);

mse_history = zeros(num_iterations, num_outputs);

for iter = 1:num_iterations

predictions = X_train * theta;

errors = predictions - Y_train;

gradients = (1/num_samples) * (X_train' * errors);

theta = theta - alpha * gradients;

mse = (1/(2*num_samples)) * sum(errors .^ 2, 1);

mse_history(iter, :) = mse;

end

mse_history_all(:, :, trial) = mse_history;

theta_all(:, :, trial) = theta;

end

avg_mse_history = mean(mse_history_all, 3);

figure;

hold on;

output_names = {'KC\_remember', 'KC\_understanding', 'KC\_application', 'KC\_analyzing', 'KC\_evaluation', 'KC\_creation'};

for j = 1:num_outputs

plot(1:num_iterations, avg_mse_history(:, j), 'LineWidth', 2, 'DisplayName', output_names{j});

end

xlabel('Iterations');

ylabel('Loss (MSE)');

title('Average Loss Function over Iterations for Each Cognitive Level (Across 100 Trials)');

legend('show');

grid on;

hold off;

avg_theta = mean(theta_all, 3);

std_theta = std(theta_all, 0, 3);

figure;

bar(avg_theta(2:end, :));

hold on;

errorbar(1:num_features, avg_theta(2:end, :), std_theta(2:end, :), 'k', 'LineStyle', 'none');

xlabel('Features');

ylabel('Coefficients');

title('Average Regression Coefficients Across 100 Trials');

set(gca, 'XTickLabel', {'HLT1', 'HLT2', 'HLT3', 'HLT4', 'FT1', 'FT2', 'FT3', 'CT1', 'CT2', 'CT3', 'CT4', 'ET1', 'ET2', 'ET3'});

xtickangle(45);

legend(output_names, 'Location', 'best');

grid on;

hold off;

avg_theta = mean(theta_all, 3);

std_theta = std(theta_all, 0, 3);

figure;

bar(avg_theta(2:end, :));

hold on;

num_features = size(avg_theta, 1) - 1;

x = repmat(1:num_features, num_outputs, 1);

x = x(:);

y = avg_theta(2:end, :);

std_dev = std_theta(2:end, :);

errorbar(x, y(:), std_dev(:), 'k', 'LineStyle', 'none');

xlabel('Features');

ylabel('Coefficients');

title('Average Regression Coefficients Across 100 Trials');

set(gca, 'XTickLabel', {'HLT1', 'HLT2', 'HLT3', 'HLT4', 'FT1', 'FT2', 'FT3', 'CT1', 'CT2', 'CT3', 'CT4', 'ET1', 'ET2', 'ET3'});

xtickangle(45);

legend(output_names, 'Location', 'best');

grid on;

hold off;

figure('Units', 'inches', 'Position', [1, 1, 6, 4]);

hold on;

output_names = {'KC\_remember', 'KC\_understanding', 'KC\_application', 'KC\_analyzing', 'KC\_evaluation', 'KC\_creation'};

colors = lines(num_outputs);

for j = 1:num_outputs

plot(1:num_iterations, avg_mse_history(:, j), ...

'LineWidth', 0.75, ...

'DisplayName', output_names{j}, ...

'Color', colors(j,:));

end

xlabel('Iterations', 'FontSize', 8);

ylabel('Loss (MSE)', 'FontSize', 8);

title('Average Loss Function over Iterations for Each Cognitive Level', 'FontSize', 8);

legend('FontSize', 8, 'Location', 'northeastoutside');

set(gca, 'FontSize', 8, 'LineWidth', 0.75);

grid on;

hold off;

print(gcf, 'avg_loss_curve_300dpi', '-dpng', '-r300');

figure('Units', 'inches', 'Position', [1, 1, 8, 5]);

bar_handle = bar(avg_theta(2:end, :), 'LineWidth', 0.75);

hold on;

ngroups = size(avg_theta(2:end, :), 1);

nbars = size(avg_theta(2:end, :), 2);

groupwidth = min(0.8, nbars/(nbars + 1.5));

for i = 1:nbars

x = (1:ngroups) - groupwidth/2 + (2*i-1) * groupwidth / (2*nbars);

errorbar(x, avg_theta(2:end, i), std_theta(2:end, i), ...

'k', 'linestyle', 'none', 'LineWidth', 0.75);

end

xlabel('Features', 'FontSize', 8);

ylabel('Regression Coefficients', 'FontSize', 8);

title('Average Regression Coefficients Across 100 Trials', 'FontSize', 8);

set(gca, 'XTickLabel', {'HLT1', 'HLT2', 'HLT3', 'HLT4', ...

'FT1', 'FT2', 'FT3', ...

'CT1', 'CT2', 'CT3', 'CT4', ...

'ET1', 'ET2', 'ET3'});

xtickangle(45);

legend(output_names, 'FontSize', 8, 'Location', 'northeastoutside');

set(gca, 'FontSize', 8, 'LineWidth', 0.75);

grid on;

hold off;

print(gcf, 'avg_coefficients_300dpi', '-dpng', '-r300');

**Interaction Term Analysis (Gradient)**

data = readtable(' ');

kc_vars = {'remember', 'understanding', 'application', ...

'analysis', 'evaluation', 'creating'};

models = {

{'CT1', 'FT1', 'FT2', 'CT1.*FT1', 'CT1.*FT2'}; % remember

{'HLT2', 'FT2', 'CT1', 'HLT2.*FT2', 'CT1.*HLT2', 'CT1.*FT2'}; % understanding

{'FT3', 'CT1', 'FT3.*CT1'}; % application

{'FT3', 'ET2', 'FT3.*ET2'}; % analyzing

{'CT3', 'FT2', 'CT3.*FT2'}; % evaluation

{'FT3', 'ET2', 'FT3.*ET2'} % creation};

alpha = 0.001;

num_iters = 2000;

n_experiments = 100;

results = struct();

for exp_num = 1:n_experiments

for i = 1:length(kc_vars)

predictors = models{i};

X = ones(height(data), 1);

for j = 1:length(predictors)

X = [X, get_variable_column(predictors{j}, data)];

end

y = data.(kc_vars{i});

theta = zeros(size(X,2), 1);

for iter = 1:num_iters

h = X * theta;

error = h - y;

gradient = (1/length(y)) * (X' * error);

theta = theta - alpha * gradient;

end

if ~isfield(results, kc_vars{i})

results.(kc_vars{i}) = zeros(n_experiments, length(theta));

end

results.(kc_vars{i})(exp_num, :) = theta';

end

end

for i = 1:length(kc_vars)

theta_mean = mean(results.(kc_vars{i}), 1);

theta_std = std(results.(kc_vars{i}), 1);

var_names = ['Intercept', models{i}];

disp(['Model: ', kc_vars{i}]);

T = table(var_names', theta_mean', theta_std', ...

'VariableNames', {'Variable', 'Mean_Coefficient', 'Std_Dev'});

disp(T);

end

function col = get_variable_column(expr, data)

if contains(expr, '.*')

parts = strsplit(expr, '.*');

var1 = strtrim(parts{1});

var2 = strtrim(parts{2});

col = data.(var1) .* data.(var2);

else

col = data.(expr);

end

end
