# Supplementary material for: Understanding dimensions of trust in AI through quantitative cognition: Implications for human-AI collaboration
Source: PLoS One. 2025 Jul 2;20(7):e0326558. doi: 10.1371/journal.pone.0326558 (PMC12221052; doi:10.1371/journal.pone.0326558)
Supplement: Attachment — Submitted filename: Response to Reviewers.doc [file pone.0326558.s008.doc]

**Reviewer #1:** This research delves into the influence of TiAI on HAC by employing the SECI model and Bloom’s cognitive taxonomy. It enriches the trust theory through highlighting the role of TiAI in knowledge collaboration and spillover. This study holds certain theoretical and practical significance，contributing to the existing body of knowledge in the relevant academic field.

Comments:

1. The paper fails to disclose the relevant experiences of the subjects in the application or study of artificial intelligence. The knowledge background of the subjects in the AI domain has the potential to exert a notable influence on the final research conclusions.

**Response:** We did not explicitly present the participants’ prior experiences in the application or study of artificial intelligence in the paper. However, we did collect this information during the study. The decision to omit it was based on the following considerations:

1. This variable was not a significant predictor in our data analysis model;
2. To streamline the manuscript and enhance readability;
3. Most students had already taken relevant courses and participated in cognitive internships before their senior year, equipping them with foundational AI knowledge.

Nevertheless, we acknowledge the reviewer’s suggestion, as prior experience and knowledge structure may theoretically influence the dependent variable. To address this, we have incorporated relevant details in the **"Sample and Data Collection" section** to strengthen the rigor of our study.

2. The data collection period of the questionnaire spanned from 2022 to 2023. However, during 2024 and 2025, there have been remarkable breakthroughs in the field of artificial intelligence, particularly in GAI. The general public's perception of AI - based tools has also undergone substantial changes. Thus, it is necessary to question whether this study still retains its timeliness and relevance?

**Response:** AI applications in the domain of GAI have indeed garnered significant attention. However, the core conclusions of this study remain both universal and timely. First, deep learning and neural network algorithms have been widely integrated into various applications since as early as 2012, and the rapid advancement of modern AI has been largely driven by the synergy of General Purpose Machine Learning (GPML), Data Generation, and Domain-Specific Knowledge Structures (Taddy, 2018). Second, although AI technology continues to evolve, the theoretical framework of AI trust remains applicable, particularly in the context of HAC and its role in fostering innovation. Furthermore, while GAI technology witnessed a breakthrough in 2024, the fundamental functionalities of AI and the mechanisms of human-AI trust have remained consistent across various application scenarios.

Additionally, our research team acknowledges the rapid evolution of AI technologies. As a result, we have addressed this consideration in the **outlook section** of the manuscript:

“Fourth, the data collection period of this study spanned from 2022 to 2023, during which GAI technology was still in its early stages. Future research should further explore the potential impact of generative AI and other emerging technologies on human-AI collaboration and AI trust dimensions, while also validating the generalizability of these findings across a broader range of industries and cultural contexts.” **(line: 565-569)**

Taddy, M. (2018). *The technological elements of artificial intelligence* (No. c14021). National Bureau of Economic Research. DOI: 10.3386/w24301

3. The subjects of this study are data sourced from financial interns. It is necessary to clarify the reasons for choosing financial interns rather than students from other disciplines. Moreover, it is essential to elucidate whether there are any close correlations between the research conclusions and the financial domain. Additionally, inquiries should be made regarding whether the final research conclusions are applicable to other fields.

**Response:** First, our sample includes not only finance interns but also students majoring in business administration. (The lack of clarity in our manuscript’s wording may have led to some misunderstanding.) Students in these disciplines typically possess strong data analysis and forecasting skills, which are increasingly valuable in today’s evolving labor market (Alekseeva et al., 2021). In particular, these skills are directly linked to critical tasks such as quantitative analysis and risk assessment in the financial sector—domains that serve as highly representative and typical cases for studying Human-AI Collaboration (HAC).

Second, we emphasize the essential role of these skills in AI applications. AI-driven financial tasks, such as quantitative analysis and risk assessment, not only require advanced data processing capabilities but also demand a deep understanding of business contexts and decision-making processes. By selecting interns from these disciplines, our study effectively captures the real-world challenges and opportunities associated with HAC in professional environments.

Finally, regarding the generalizability of our findings, while this study focuses on the financial sector, its core conclusions—particularly those concerning skill complementarity and task allocation in human-AI collaboration—extend beyond finance. Other industries, including healthcare, law, and education, are also experiencing rapid AI integration and shifts in skill demands. Therefore, our findings provide valuable insights applicable to a broad range of fields.

To address this consideration, we have refined the **Sample and Data Collection** section of the manuscript as follows:

“The selected interns specialize in finance and business administration, fields that emphasize critical professional skills such as data analysis and forecasting, as well as strong collaboration abilities. The cultivation and acquisition of these skills significantly impact the labor market during its current transformation [14,43]. Moreover, the AI applications associated with these skills—such as quantitative analysis and risk assessment—are highly representative in HAC scenarios.” **(line: 334-340)**

Alekseeva L, Azar J, Giné M, Samila S, Taska B. The demand for AI skills in the labor market. Labour Economics. 2021;71: 102002. doi:10.1016/j.labeco.2021.102002

**Reviewer #2: Hi Author(s),**

Thank you for submitting your work to PLOS ONE. The paper offers some insights for the field of human-AI literature. Please find my feedback below, which I hope will help you enhance your work.

1. **Abstract**

The abstract lacks clarity and a comprehensive overview, making it difficult to grasp the paper’s main contributions. I recommend restructuring it to clearly outline:
1. The research problems being addressed.
2. The objectives of the study.
3. The methodology employed (e.g., data collection techniques).
4. The key findings of the research.
5. The implications for both practice and theory.
6. Suggestions for future research.
This structured approach will provide readers with a concise yet thorough understanding of the paper’s significance.

**RESPONSE:** We have further refined the summary and followed the structure you provided.

The details are as follows:

“Human-AI collaborative innovation relies on effective and clearly defined role allocation, yet empirical research in this area remains limited. To address this gap, we construct a cognitive-level AI trust framework to describe and explain its interactive mechanisms in human-AI collaboration, specifically its complementary and inhibitive effects. Specifically, we examine the alignment between AI trust and different cognitive levels, identifying key drivers that facilitate both lower-order and higher-order cognition through AI. Furthermore, by analyzing the interactive effects of multidimensional AI trust, we explore its complementary and inhibitive influences. We collected data from finance and business administration interns using surveys and the After-Action Review (AAR) method and analyzed them using the gradient descent algorithm. The findings reveal a dual effect of AI trust on cognition: while functional and emotional trust enhance higher-order cognition, the transparency dimension of cognitive trust inhibits cognitive processes. These insights provide a theoretical foundation for understanding AI trust in human-AI collaboration and offer practical guidance for university-industry partnerships and knowledge innovation.”

2. **Introduction**

The introduction does not clearly articulate the paper's motivation and would benefit from a more comprehensive literature review. It is recommended that each paragraph focus on a specific research gap so that, collectively, they build a coherent narrative demonstrating how your research addresses these gaps. The authors should also identify the research questions in the introduction part.
Additionally, the paper overlooks two seminal works in AI by Huang and Rust (2021, 2024). These studies present an AI framework that spans from mechanical intelligence to cognitive reasoning (thinking intelligence) and affective understanding (feeling intelligence). Incorporating these references could provide critical context and strengthen the discussion on AI trust.

**Response:** We appreciate this recommendation and have made substantial revisions based on the feedback from both Reviewer 2 and Reviewer 3. Specifically, we have implemented the following improvements:

Structural Adjustments: We have reorganized the introduction to establish a clearer and more coherent narrative. The revised structure now explicitly addresses the background, research significance, identified gaps, specific objectives, and broader implications of our study.

Emphasis on Research Gaps: We have strengthened the discussion on AI trust research gaps, focusing on the following key aspects:

The underlying mechanisms of human trust in AI remain insufficiently understood.

Most existing studies lack a multidimensional perspective on AI trust interactions.

There is a notable absence of empirical evidence in research on human-AI coexistence theories, particularly concerning role allocation in collaborative settings.

Justification for Cognitive Hierarchies: We have elaborated on why cognitive hierarchies serve as an effective framework for interpreting human-AI collaboration and AI trust. Additionally, we highlight the advantages of this approach in addressing the identified research gaps.

Incorporation of Seminal Works: We have carefully reviewed and integrated insights from Huang and Rust (2018, 2021, 2024), as well as Okamura and Yamada (2020). Their discussions on AI’s progression from mechanical intelligence to cognitive reasoning and affective understanding have provided valuable context for our study. In particular, their findings on threshold effects in AI trust have reinforced the need for further exploration of multidimensional AI trust interactions, particularly in the context of human-AI role allocation.

These refinements significantly enhance the clarity and rigor of our introduction, ensuring that our study is well-positioned within the broader AI trust literature.

3. **Literature Review**

Upon reviewing the section, I found the structure of the writing somewhat difficult to follow. It is not clear what has been accomplished in the fields of cognitive quantification, human-AI interaction and AI trust, nor is it evident how your research addresses the existing gaps from a theoretical perspective.
I recommend that the authors:
• Clarify the Theoretical Framework: Clearly identify and highlight the key theories that underpin the research. A critical synthesis of previous findings should be provided to illustrate how your work builds upon and extends existing knowledge. Kindly take a look at previously published work at Plos One by Okamura & Yamada, 2020.
• Enhance Table 1: The current presentation in Table 1 is vague. It should explicitly state the findings from the reviewed papers and detail how these findings contribute to or contrast with the contributions of your study.
• Develop a Conceptual Model: The paper would benefit significantly from the inclusion of a conceptual model and the formulation of hypotheses. This would help in showcasing the relationships between the key constructs of AI trust and in framing the research contributions more coherently.
Incorporating these suggestions will help clarify your research direction and enhance the overall coherence and impact of the paper.

**Response:**

The work of Okamura & Yamada (2020) has provided us with significant insights, particularly regarding the potential threshold effects of AI trust—where exceeding or falling below a certain level may either enhance or inhibit trust. However, our research focuses on the multidimensional nature of AI trust. Given this perspective, our team believes it is essential to empirically validate the interactive effects of AI trust across multiple dimensions. This multidimensional interaction study does not contradict our previous research; rather, it further clarifies and expands on the dynamics of harmonious human-AI relationships.

Regarding the summary of AI trust research in Table 1, we have moved the original Table 1 to the supplementary materials and provided a more detailed discussion of relevant AI trust studies. In fact, we initially offered an in-depth interpretation of Table 1 within the manuscript. However, we opted to streamline this section for a specific reason: *PLOS ONE* has a strong empirical and methodological orientation, with a writing style that emphasizes conciseness and clarity. Therefore, in the *Revised Manuscript with Track Changes*, we have made the following adjustments: The original *Table 1. TiAI types and constructs* has been relocated to *S1. Table AI trust research literature collation*. Additionally, we have expanded our review of recent AI trust research, particularly studies published since 2022, in the *AI Trust Dimensions* section.

To further clarify our research objectives and contributions to addressing existing gaps, we have restructured the *Theoretical Foundations* section.

Reviewer 2 suggested: *"Develop a Conceptual Model: …………more coherently."* Instead of constructing a predefined conceptual model, we have chosen to refine and elaborate on our research objectives. The reason for this approach is that the relationship between AI trust and cognitive hierarchies remains unclear, and we aim to avoid imposing any premature assumptions. In the *Summary of AI Trust Research* (lines 227–249), we have revised and articulated our research objectives as follows:

- **Unveil the trust-cognition matching mechanism:** Investigate how different types of AI trust allocate tasks between lower-order cognition (remembering, understanding, applying) and higher-order cognition (analyzing, evaluating, creating).
- **Identify key influencing factors:** Determine which AI trust types play a dominant role in shaping different cognitive levels within HAC.
- **Analyze interactive effects:** Hypothesize that different AI trust types exhibit interactive effects and propose a human-AI role allocation strategy based on complementarity and inhibition.

These revisions strengthen the theoretical foundation of our study and align it more closely with the empirical focus of *PLOS ONE*.

4. **Methodology**

I can see the authors indicated the rationale behind not applying for Ethical Approval. However, every research involving human participants always requires ethical approval no matter what fields they are focusing on. The role of reviewers is to provide feedback on your work, so I would not touch that aspect; the task is left for the handling Editor.

**Response:**

We sincerely apologize for not providing the necessary approval documentation for the human subject survey earlier. This research was conducted as part of a university-level project at Wuhan Technology and Business University (Project No.: XJ2023000501). The study was initially planned in 2022, and in 2023, we conducted a survey among interns at Wuhan Technology and Business University, for which we obtained the relevant approvals.

However, we would like to clarify an important point: In Wuhan, institutional ethics committees, such as those at Wuhan University and Huazhong University of Science and Technology, primarily review research involving medical and animal studies. Our study falls under the JEL classifications O31, O32, D83, and M15, which are outside the scope of local ethics committee reviews.

That said, our research was approved by the Research Department of Wuhan Technology and Business University, which is responsible for overseeing and regulating research surveys conducted within the university.

To ensure compliance with research ethics and institutional regulations, we have now submitted the relevant documentation as evidence of our research approval, including:

- **Approval for Research Survey on Interns from Wuhan Technology and Business University**
- **Notification of Project Approval for the 2023 Scientific Research Program of Wuhan Technology and Business University**
- **Informed Consent Form**

The use of the gradient descent method in this study raises important questions regarding its necessity and appropriateness. While the paper mentions that gradient descent offers advantages like computational efficiency and handling nonlinear data, it does not provide a clear justification for why this specific method was chosen over more conventional regression techniques. Given the nature of your data and research objectives, is the complexity of gradient descent truly required? Traditional statistical methods such as linear or logistic regression, which are easier to interpret and widely accepted in similar research contexts, might suffice.

**Response:**

Our research aims to explore the **matching mechanism between the multidimensionality of AI trust and different cognitive levels**. To achieve this, we construct a framework for AI trust, which consists of **four major dimensions and fourteen specific items**. We deliberately chose not to aggregate these items into broader categories, as our primary goal is to establish an **efficient and clear human-AI role allocation**.

Accordingly, the construction of our model must meet the following criteria:

1. **Optimization** – Identifying the optimal value for role allocation.
2. **Multidimensionality** – Addressing the complexity of AI trust.
3. **Non-linearity** – Capturing the intricate relationships between AI trust and cognition.
4. **Efficiency** – Ensuring computational feasibility and effectiveness.

In response to **Reviewer 2’s suggestion**, we acknowledge its importance and have incorporated it into our manuscript under **Methodology—Establishing the Impact of AI Trust on Cognition**. In this section, we elaborate on both the **advantages and limitations of the gradient descent method**, explain why we adopted this approach, and describe the measures taken to **overcome its drawbacks**.

The explanation of the independent (TiAI dimensions) and dependent variables (cognitive levels) is superficial. While constructs like human-like trust, functionality trust, cognitive trust, and emotional trust are mentioned, their operational definitions, measurement scales, and specific indicators are not clearly detailed. This lack of clarity makes it difficult to assess the validity and reliability of the variables used. As indicated in the study that developed a trust scale for AI trust, I would suggest the authors take a look at EFA and CFA.

**Response:**

First, we acknowledge that in our original manuscript, we described the relationship between AI trust and cognition in terms of independent and dependent variables. In the **Revised Manuscript with Track Changes**, we have provided further clarification on this matter. We argue that AI’s influence on cognition is best understood as a **matching relationship**, which is inherently **multidimensional and complex**.

Second, we have revised **Table 2. Questionnaire Structure and Item Composition** and **S3. Table Quantitative Cognitive** by defining and explaining each variable in greater detail.

Finally, regarding the **development of the measurement scales**, we have conducted comprehensive validation analyses. In **S2. Table Reliability, Validity, and Convergence of AI Trust Questionnaire Items** and the **Quantification of Cognitive Taxonomy** section, we have examined key statistical metrics, including **KMO, reliability (Cronbach’s alpha), AVE, CR, and total variance explained**. Additionally, we deliberately chose **not to aggregate variables** to preserve the structural integrity of the data. As a result, in our **CFA**, we did not assess model fit indices such as **CFI or TLI**. Instead, in our **gradient descent approach**, we evaluated model stability using **MSE, parameter tuning, and multiple experimental trials**.

The methodology depends on self-reported questionnaire data to assess trust in AI and cognitive behaviours. This shows potential biases, including social desirability bias and self-perception inaccuracies. How could the authors reduce those biases during the data collection process? We need a very clear justification here.

**Response:**

We employed not only traditional questionnaire-based AI trust measurements (e.g., Likert scales) but also **task-based scenario simulations**, where participants were required to complete specific tasks while their AI usage behaviors were recorded under different conditions. This approach allowed us to capture **real-world interactions with AI trust**, rather than relying solely on self-reported data.

When assessing cognitive levels through **AAR surveys**, we integrated both **performance outcomes and test results** with AI trust questionnaire measures. For instance, in **evaluative tests**, we used **ChatGPT** to analyze participants’ **comments and suggestions** on AI-generated recommendations to assess **affective trust**, filtering out inconsistent responses.

Furthermore, we conducted a **professional skills assessment** for each participant. One of the key objectives of this study was to **minimize subjective bias** in participant responses.

5. Results and Discussion
   I have no further comment regarding the results section till the issues in the methodology section are resolved.
   The discussion should tie the results back to the original research objectives or gaps identified in the literature review. For example, while the study identifies different dimensions of AI trust and their impact on cognitive processes, it does not clearly articulate how these findings advance our understanding of human-AI collaboration (HAC) or contribute to existing theories.

**Response:**

Reviewer 2 did not provide specific suggestions for our study in this section. However, we have made revisions based on the suggestions from Reviewer 3.

**Reviewer #3:** Thank you for the opportunity to review the paper titled “Understanding AI Trust Dimensions through Quantitative Cognition: Implications for Human-AI Collaboration’’.

This is an interesting study and I commend the authors on taking the road least travelled. I have some comments to improve the manuscript below.

First, I believe that the abstract is not well-structured. I think the research aims are not stated frankly and specifically, making it difficult for readers to decide to read the full article. The research method is quite vague when lacking statement about the number of samples and scope. Originality and Values are summarized from the results of the article, so it clearly indicates the implications for both academic and practical circles.

**Response:**

I have revised the abstract according to the following structure:

1. The research problem being addressed.
2. The research objectives.
3. The methodology employed (e.g., data collection techniques).
4. The key findings of the study.
5. The practical and theoretical implications.
6. Recommendations for future research.

The introduction provides some foundation but could benefit from further analysis, structure, and clarity to enhance its impact. It is recommended that the text be divided into more specific paragraphs: (1) Provide an overview of the context of the study, (2) Outline the importance of the study, (3) Highlight the gap in the existing literature, (4) State the specific objectives and implications of the study. Especially the paragraph about the gaps of the article should be more carefully cared for because I do not see the attractiveness and urgency of the gaps that you are simply listing. I recommend that you add to the arguments or calls to strongly emphasize the need for the filling of each gap you mentioned.

**Response:**

We accept this suggestion. Based on the recommendations of Reviewer 3 and Reviewer 2, we have made the following adjustments to the introduction:

First, we have restructured it following Reviewer 3’s suggested framework: background, research significance, research gap, specific objectives, and impact.

Second, we have thoroughly addressed the research gap in AI trust, emphasizing the following points: (1) the mechanisms underlying human trust in AI remain insufficiently understood; (2) most existing studies lack a multidimensional interactive perspective; and (3) empirical research on human-AI coexistence theory is scarce, with limited exploration of role allocation between humans and AI.

Third, we have explained why cognitive levels provide a valuable framework for interpreting human-AI collaboration and AI trust, highlighting the advantages of this approach in bridging the identified research gap.

Finally, we carefully reviewed the works of Huang and Rust (2018, 2021, 2024) and Okamura & Yamada (2020), which provided significant insights. For instance, the threshold effect in AI trust inspired us to further investigate multidimensional interactions in AI trust to better explain human-AI role allocation.

Huang M-H, Rust RT. Engaged to a Robot? The Role of AI in Service. Journal of Service Research. 2021;24: 30–41. doi:10.1177/1094670520902266

Huang M-H, Rust RT. The Caring Machine: Feeling AI for Customer Care. Journal of Marketing. 2024;88: 1–23. doi:10.1177/00222429231224748

Okamura K, Yamada S. Adaptive trust calibration for human-AI collaboration. Lv C, editor. PLoS ONE. 2020;15: e0229132. doi:10.1371/journal.pone.0229132

The theoretical foundations could benefit from a more comprehensive exploration of an updated literature review to align with recent advancements in the field. Only a few adequate reviews of recent literature are used in this research. You can consider changing literature that is older than 2022, most of the documents you use are quite old (citation 13-42) and this reduces the reliability of a study in a fast-moving field like AI. Please check and add more discussions from the most recent scholars.
**Response:**

We accept this suggestion and have made the following improvements. Specifically, in the ***Theoretical Foundations*** section, we have reorganized the literature on AI trust and AI’s role in innovation collaboration. We have removed studies that are not particularly relevant or do not significantly contribute to the core theme of our paper. However, we have retained certain milestone works that are foundational to AI trust research.

Demographic information should be presented in detail into the table form in the sample section.

**Response:**

We accept the suggestion and have made improvements accordingly. Specifically, in the *Sample and Data Collection* section, we have added Table 1 (*Distribution of Demographic Characteristics of Respondents (N=408)*) to provide a detailed description of the demographic data.

The presentation of results in the paper aligns with the analysis performed, providing clarity in reporting findings.
The discussion section, while commendable, is seen as ambiguous and lacking depth. The discussion part is where you delve into the meaning, significance, and relevance of your findings. It should be focused on discussing and evaluating what you discovered, demonstrating how it pertains to your literature review and research objectives, and presenting an argument in favor of your ultimate conclusion. How do you interpret these findings, and how do they compare to previous studies in the field? This study should compare the novelty to current and previous research for each conclusion, and then interpret the research piece in a real-world perspective.
**Response:**

We accept the suggestion and have made the following improvements:

**Refinement of Research Objectives in the Theoretical Foundations Section**

In the *Theoretical Foundations - Summary of AI Trust Research* section, we have revised and clarified the research objectives as follows:

1) **Unveil the trust-cognition matching mechanism**: Investigate how different types of AI trust allocate tasks between lower-order cognition (remembering, understanding, applying) and higher-order cognition (analyzing, evaluating, creating).
2) **Identify key influencing factors**: Determine which AI trust types play a dominant role in shaping different cognitive levels within HAC.
3) **Analyze interactive effects**: Hypothesize that different AI trust types exhibit interactive effects and propose a human-AI role allocation strategy based on complementarity and inhibition.

**Enhancements to the Discussion Section**

To align with the refined research objectives, we have improved the discussion as follows:

1) Analyzed and discussed the matching relationship between AI trust and different cognitive levels, distinguishing between AI trust’s effects on higher-order and lower-order cognition.
2) Examined parameter tuning and robustness in the gradient descent method.
3) Explored the multidimensional interactions of AI trust.

The conclusion section should be restructured as following: theoretical and practical implications, then limitations and future research. The paper attempts to identify implications for theory but it’s still shallow, which needs more justifications and aligns with the results.

**Response:**

We have expanded and refined the conclusion and contribution sections, specifically adding and optimizing the following aspects:

1) **Theoretical Contributions** – Further elaboration on the trust-cognition matching mechanism in AI, exploring human-AI role allocation in collaboration, and addressing gaps in multidimensional AI trust interaction research.
2) **Practical Contributions** – Proposal of an AI trust-based intelligent task allocation strategy to guide real-world applications.
3) **Limitations and Future Directions** – Discussion of the study’s limitations and suggestions for future research directions.

For details, please refer to *lines 505-574*.

I appreciate your dedication to this research topic, and I believe that addressing these points will contribute significantly to refining the manuscript. While I acknowledge the potential significance of your work, I recommend the major revisions of the manuscript in its current form. I am confident that your revisions will strengthen the scholarly merit of your contribution.
